# The Needs of Bachelors

**Published:** 1888-07-07

**Authors:** Wm. M. Rains


### THE NEEDS OF BACHELORS.

I see by The Hospital that you have noticed what I mentioned, viz., "The Miseries of a Bachelor," in your well-written article called, "The Nursing of Bachelors." But I must object (excuse my saying so) to your saying that it is only poverty that prevents the bachelor becoming a Benedict. It has often occurred to me that men of limited means, residing in apartments or chambers, after paying, say 20*s.* a-week for their rooms, and perhaps extras, and the doctor's daily visits, amounting to another guinea or more—how can such a man afford a nurse whom he must pay another guinea, besides her board and all other expenses? A man with an income, say of £150 a-year, cannot do it. I know, and no doubt you do, of such cases; and if such men only knew where to procure a trustworthy woman, different from the regular lodging-house charwoman, it would be a great boon. Your paper is not, to my idea, sufficiently known to the public; it was by chance I first met it, and not one of my six friends, to whom I sent copies last week, had ever heard of it. I have again sent a few copies of this week's number. I do think, if you will occasionally notice in your paper "A Bachelor's wants," that it will do good. As you justly say, there are nurses for the poor and the rich, but the middle strata are left out.

Wm. M. Rains.

